# Analysis of risk factors for negative emotions in patients with thyroid nodules: A cross-sectional study

**DOI:** 10.1097/MD.0000000000040548

**Published:** 2024-11-22

**Authors:** Mengxuan Wang, Shuo Wang, Guoshan Yuan, Mingzhou Gao, Jieqiong Wang, Zhenhan Chu, Lv-Ning Ren, Dongmei Gao

**Affiliations:** a College of Traditional Chinese Medicine, Shandong University of Traditional Chinese Medicine, Jinan, Shandong Province, China; b College of Medical Information Engineering, Shandong University of Traditional Chinese Medicine, Jinan, Shandong Province, China; c Innovation Institute of Chinese Medicine and Pharmacy, Shandong University of Traditional Chinese Medicine, Jinan, Shandong Province, China.

**Keywords:** anxiety, depression, negative life events, risk factors, thyroid nodules

## Abstract

Investigations have indicated that there is a correlation between thyroid nodules and patients’negative emotions. Nevertheless, the risk factors contributing to the development of negative emotions in thyroid nodule patients remain unidentified. This cross-sectional study recruited 150 patients diagnosed with thyroid nodules through ultrasound examination from January 2022 to January 2023 at Jinan Central Hospital, the Second Affiliated Hospital of Shandong First Medical University, and Qingyun County Maternal and Child Health Hospital as the case group, which were categorized based on their levels of anxiety and depression. Simultaneously, 150 individuals with normal thyroid ultrasound findings were selected as the control group. The researchers chose a self-administered general information questionnaire and 6 psychological scales as the assessment tools for the patients. SPSS 26.0 was used to analyze the risk factors for negative emotions in patients with thyroid nodules. The scores of the self-rating anxiety scale were higher in the case group than in the control group (40.90 ± 9.490 vs 38.37 ± 6.836, *P* = .028), as were the scores of the self-rating depression scale (44.35 ± 9.180 vs 41.48 ± 8.297, *P* = .004). There is a positive correlation between thyroid nodules and degrees of anxiety and depression (*R* = 0.176, *P* = .002; *R* = 0.206, *P* = .000). The results of the binary logistic regression analysis revealed that both fatigue state (*P* = .013) and negative life events (*P* = .001) exerted independent effects on anxiety, whereas negative life events (*P* = .002) had independent effects on depression. This study enhances the understanding of the relationship between thyroid nodules and anxiety and depression. It reveals that thyroid nodules are associated with negative emotions and that negative life events have a significant influence on anxiety and depression in these individuals, which may contribute to the development of strategies for preventing and treating thyroid nodules in clinical practice.

## 1. Introduction

Thyroid nodules are mass structures with one or more histological abnormalities within the thyroid parenchyma, resulting from a diverse range of causes.^[1]^ Thyroid nodules (TNs) are widely recognized as a prevalent disorder within the endocrine system. TNs have a tendency to exhibit either benign or malignant characteristics, with approximately 10% to 15% of cases demonstrating malignancy.^[2]^ In severe instances, TNs may cause sensations of a foreign body and pressure, along with mental and emotional abnormalities. These symptoms can significantly impact the daily lives and professional activities of individuals affected by TNs, imposing both physical and psychological burdens on patients and their families.^[3]^ The detection rate of TNs has experienced a significant increase in recent years due to the increased knowledge of thyroid illnesses among individuals and advancements in ultrasound technology.^[4]^ Additionally, there is a favorable correlation between the incidence of TNs and age.^[5,6]^ Approximately 7% to 15% of TN cases can develop into thyroid cancer^.[1]^ According to statistics from 2020,^[7]^ thyroid cancer ranks as the ninth most prevalent cancer globally. Furthermore, the prevalence of thyroid cancer is starting in high-income nations and extending to low- and middle-income countries. Consequently, the use of early health management strategies for TNs has the potential to mitigate the occurrence of thyroid cancer to a certain degree. Hence, it is imperative to manage the risk factors associated with TNs.

Anxiety and depression frequently co-occur within the realm of psychiatric diseases.^[8]^ These conditions can lead to impaired physical functioning and, in severe cases, even suicide, contributing to the overall load on global healthcare systems.^[9]^ A survey conducted in China found that anxiety and depression have lifetime prevalence rates of 7.6% and 3.4%, respectively.^[10]^ Traditional Chinese medicine holds that TNs are closely related to negative emotions,^[11,12]^ and the use of methods to soothe the liver, regulate qi, and relieve depression has an overall effective rate of up to 75% in treating TNs, effectively enhancing the clinical indicators of thyroid function.^[13]^ Modern medicine has also provided evidence indicating a strong correlation between thyroid disorders, anxiety, and depression.^[14–16]^ Furthermore, situations of stress can activate psychological stress responses, leading to disruptions in the regulation of the hypothalamus–pituitary–adrenal (HPA) and hypothalamus–pituitary–thyroid (HPT) axis, consequently impacting the functioning of the thyroid gland^.[17]^ However, the majority of studies have concentrated on the relationship between TNs and anxiety and depression. This study aims to further explore the risk factors for negative emotions in TNs patients from a psychological perspective, building on previous research.

## 2. Materials and methods

### 2.1. Study design and sample

This cross-sectional study selected patients who sought treatment at Jinan Central Hospital, the Second Affiliated Hospital of Shandong First Medical University, and Qingyun County Maternal and Child Health Hospital between January 2022 and January 2023 as subjects.

Inclusion criteria: (1) age 18 and above; (2) willingness to participate in this study and consent to cooperate in filling out the questionnaire; (3) consciousness and capability of making independent judgments; (4) having thyroid ultrasound examination data.

Exclusion criteria: (1) people who did not agree to participate in this study or whose questionnaires were incompletely filled out; (2) patients with mental disorders or others unable to cooperate; (3) pregnant and lactating women; (4) patients who have undergone thyroidectomy or microwave ablation; (5) individuals with concurrent thyroiditis, thyroid cancer, or other thyroid-related diseases.

**Figure 1.**
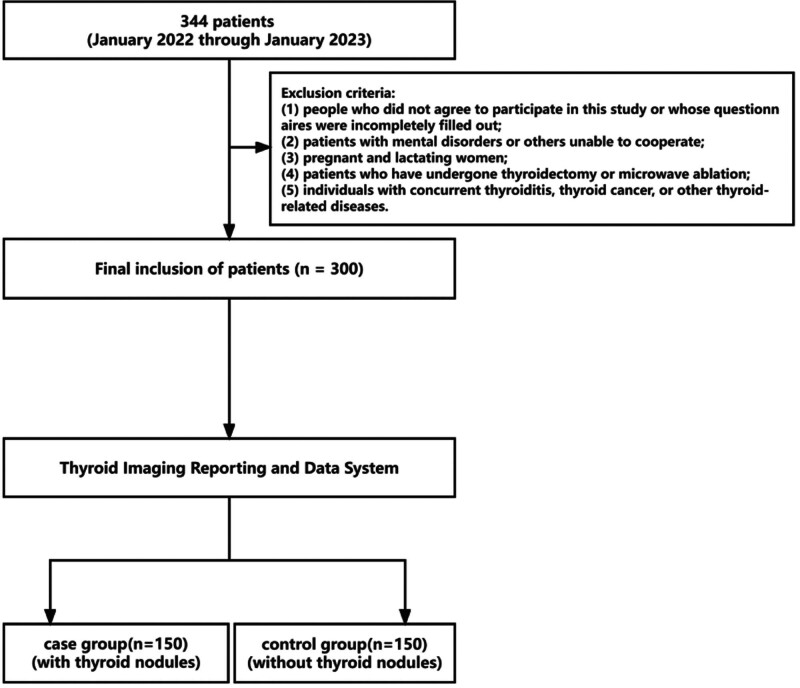
A flowchart showing the study procedure.

A cohort of 344 individuals was enrolled. Fourty-four participants were excluded from the study: 13 individuals had incomplete questionnaires, 3 individuals had undergone microwave ablation, and 28 individuals had been diagnosed with additional thyroid illnesses. A total of 300 patients were included in the study. According to the Thyroid Imaging Reporting and Data System,^[1]^ they were divided into 150 patients in the case group (with thyroid nodules) and 150 patients in the control group (without thyroid nodules) (Fig. 1). Informed consent was obtained from all participants.

### 2.2. Data collection

General information: self-administered scale including age, sex, body mass index, marital status, education, family history, the presence of thyroid-related disorders, and clinical data (such as number of nodules, echo characteristics, composition, borders, aspect ratio, etc).

Self-rating anxiety scale (SAS): Assessing the patient’s anxiety levels,^[18]^ the SAS explores emotional symptoms based on diagnostic criteria outlined in major American psychiatric literature. The SAS score is divided based on a standard of 50 points: 50–59 indicates mild anxiety, 60–69 indicates moderate anxiety, and ≥70 indicates severe anxiety.

Self-rating depression scale (SDS): To assess patients’ depressive levels,^[19]^ the selection of items in the SDS is based on factor analysis studies of depressive symptoms. The SDS scores were divided according to a standardized score of 50 as a cutoff point: 50–59 indicates mild depression, 60–69 indicates moderate depression, and ≥70 indicates severe depression.

Eysenck personality questionnaire revised short scale: This scale is used to assess the personality characteristics of patients.^[20]^ There are 4 subscales with a total of 48 items: psychoticism (P), extraversion (E), neuroticism (N), and the Lie scale (L). Each subscale has 12 items. Higher scores on each subscale correspond to more pronounced personality traits in that dimension.

Pittsburgh sleep quality index: Patients’ sleep quality was evaluated.^[21]^ The 7 categories on the scale—sleep quality, sleep duration, sleep onset latency, sleep efficiency, sleep disturbances, daytime functioning, and sleeping medication—evaluate the overall sleep situation of the patient over the past month. Judges consider a total score of ≥8 as indicative of poor sleep quality.

Fatigue scale: This scale is used to assess the level of fatigue in patients.^[22]^ It consists of 14 items, with 8 items related to physical fatigue and 6 items related to mental fatigue. A total score of ≥3 is indicative of the presence of fatigue.

Life events scale: This survey is used to assess negative life events experienced by patients in the past year.^[23]^ The content includes 38 items related to various aspects of life, including family, work, education, social, and others. Each item has 2 options: “yes” and “no.” Responding “yes” to an item is scored as 1 point, while responding “no” is scored as 0 points. A higher score indicates a greater number of negative life events experienced.

### 2.3. Statistical analysis

Data analysis was conducted using SPSS 26.0 (IBM Corp., Armonk, NY) statistical software. The mean ± standard deviation was used to represent continuous variables, while percentages were used to represent categorical variables. The Mann–Whitney *U* test was utilized to compare continuous variables, while the Chi-square test was implemented for the comparison of categorical variables. Spearman correlation analysis was used to analyze the correlation between thyroid nodules and levels of anxiety and depression. Variables with a univariate *P* < .05 were included in the multivariate analysis. Binary logistic regression was employed to evaluate the characteristics that influence anxiety and depression among individuals with TNs. The assignment of anxiety and depression was defined as (0 = No, 1 = Yes) in the final model to avoid violating the principle of excluding linearly codependent variables. ORs and corresponding 95% CIs were estimated based on the multivariable model. All tests were 2-sided, and a *P*-value < .05 was considered statistically significant.

## 3. Results

### 3.1. General information

A total of 300 subjects, comprising 51 males (17.0%) and 249 females (83.0%), were enrolled in this study. The average age of the participants was 43.15 ± 10.714 years. The prevalence rates of anxiety and depression in the overall sample were found to be 15.0% and 23.7%, respectively. The participants were categorized into case and control groups based on the Thyroid Imaging Reporting and Data System (Table 1).

**Table 1:** General information.

Variable	Case group n (%), n = 150	Control group n (%), n = 150
Gender		
Male	19 (12.7)	32 (21.3)
Female	131 (87.3)	118 (78.7)
Age (years)	41.37 ± 10.456	44.92 ± 10.708
20–39	46 (30.7)	76 (50.7)
40–59	93 (62.0)	65 (43.3)
≥60	11 (7.3)	9 (6.0)
Marriage		
Married	139 (92.7)	120 (80)
Others	11 (7.3)	30 (20)
Level of education		
High school or below	52 (34.7)	41 (27.3)
Secondary and tertiary	42 (28.0)	64 (42.7)
College degree or above	56 (37.3)	45 (30.0)

### 3.2. Correlation analysis of thyroid nodules, anxiety and depression

**Figure 2.**
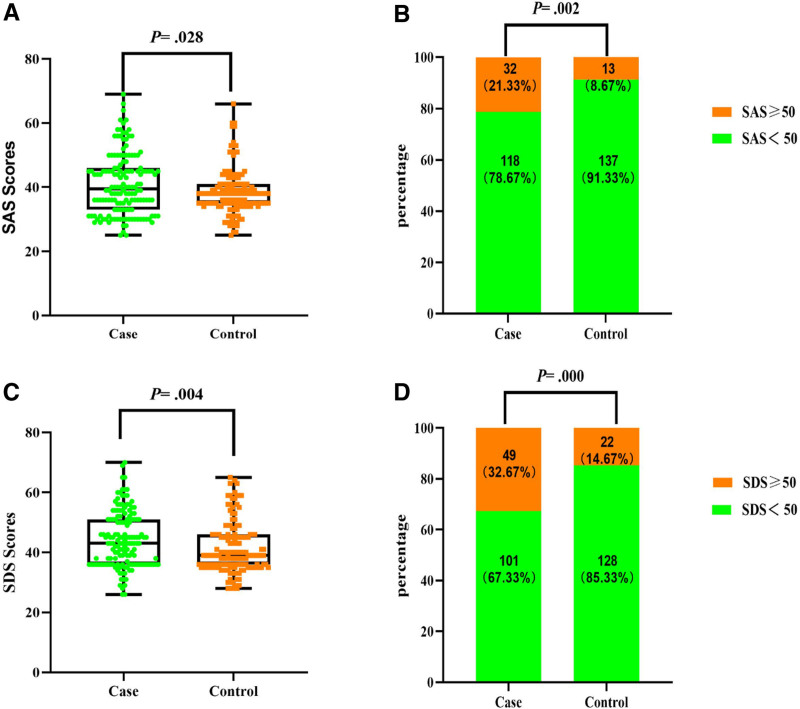
Anxiety and depression status in case and control groups. (A) SAS scores of case and control group. (B) Composition of subjects with different SAS scores in case and control group. (C) SDS scores of case and control group. (D) Composition of subjects with different SDS scores in case and control group. (A, C) Wilcoxon–Mann–Whitney *U* test. (B, D) Chi-square test. SAS = self-rating anxiety scale, SDS = self-rating depression scale.

As shown in Figure 2, the scores of SAS were higher in the case group than in the control group (40.90 ± 9.490 vs 38.37 ± 6.836, *P* = .028), as were the scores of SDS (44.35 ± 9.180 vs 41.48 ± 8.297, *P* = .004). The number of individuals with anxiety was significantly higher in the case group (21.33% vs 8.67%, *P* = .002), and the number of individuals with depression was also significantly higher in the case group (32.67% vs 14.67%, *P* = .000). Patients diagnosed with thyroid nodules have a higher rate of anxiety and depression. Furthermore, the results of the Spearman correlation showed a positive correlation between TNs and the degree of anxiety and depression (*R* = 0.176, *P* = .002; *R* = 0.206, *P* = .000; Table 2).

**Table 2:** Spearman correlation among the patients with thyroid nodules and the degree of anxiety and depression.

Variable	Case group (n = 150)	Control group (n = 150)	r	*P*
Anxiety degree				
No	118 (78.7%)	137 (91.3%)	0.176	.002
Mild	26 (17.3%)	10 (6.7%)		
Moderate	6 (4.0%)	3 (2.0%)		
Depression degree				
No	101 (67.3%)	128 (85.3%)	0.206	.000
Mild	40 (26.7%)	16 (10.7%)		
Moderate	8 (5.3%)	6 (4.0%)		
Severe	1 (0.7%)	0 (0.0%)		

### 3.3. Univariate analysis of factors affecting SAS and SDS scores in patients with TNs

As demonstrated in Tables 3 and 4, the findings of this study indicate that level of education (*P* = .037; *P* = .005), fatigue status (*P* = .043; *P* = .042) and negative life (*P* = .001; *P* = .002) were common risk factors for SAS/SDS scores. The age range of 20 to 39 years (*P* = .025) and thyroid nodule boundary (clear or not) (*P* = .022) were identified as risk factors for SAS. Furthermore, risk factors for SDS were considered to be body mass index (*P* = .042) and personality type (Phlegmatic) (*P* = .012).

**Table 3:** Univariate analysis of self-rating anxiety scale scores in patients with thyroid nodules.

Variables	SAS < 50 (No anxiety, n = 118, %)	SAS ≥ 50 (Anxiety, n = 32, %)	*χ*^2^/*Z*	*P*
Gender				
Male	15 (10.0)	4 (2.7)	0.001	.975*
Female	103 (68.6)	28 (18.7)		
Age (years)	45.48 ± 10.795	42.84 ± 10.28	‐1.274	.203†
20–39	31 (20.7)	15 (10.0)	5.062	.025*^,^‡
40–59	77 (51.3)	16 (10.7)	2.486	.115*
≥60	10 (6.7)	1 (0.6)	0.419	.517*
BMI (kg/m^2^)	22.76 ± 3.280	22.24 ± 4.616	‐1.954	.051*
Marriage				
Married	109 (72.7)	30 (20.0)	0	1*
Others	9 (6.0)	2 (1.3)		
Level of education				
High school or below	44 (29.3)	8 (5.3)	1.678	.195*
Secondary and tertiary	35 (23.3)	7 (4.7)	0.757	.384*
College degree or above	39 (26.0)	17 (11.3)	4.336	.037*^,^‡
EPQ-RSC				
Melancholic	30 (20.0)	5 (3.3)	1.351	.245*
Phlegmatic	20 (13.3)	6 (4.0)	0.057	.811*
Choleric	37 (24.7)	15 (10.0)	2.677	.102*
Sanguine	31 (20.7)	6 (4.0)	0.766	.381*
Number of nodules				
Solitary nodule	38 (25.3)	7 (4.7)	1.279	.258†
Multiple nodules	80 (53.3)	25 (16.7)		
Nodular echogenicity				
Hypoechoic	67 (44.7)	22 (14.7)	1.495	.221†
Non-hypoechoic	51 (34.0)	10 (6.6)		
Components of nodules				
Mainly solid	61 (40.6)	16 (10.7)	0.029	.865†
Mainly cystic	57 (38.0)	16 (10.7)		
Boundary				
Clear boundary	99 (66.0)	21 (14.0)	5.254	.022†^,^‡
Unclear boundary	19 (12.7)	11 (7.3)		
Aspect ratio				
<1	94 (62.7)	29 (19.3)	2.050	.152†
>1	24 (16.0)	3 (2.0)		
Sleeping disorders	6.47 ± 3.625	6.28 ± 3.54	-0.443	.665†
Yes	21 (14.0)	11 (7.3)	0.003	.960*
No	78 (52.0)	40 (26.7)		
Fatigue	6.37 ± 3.961	6.88 ± 3.098	-0.900	.368†
Yes	87 (58.0)	29 (19.3)	4.100	.043*^,^‡
No	31 (20.7)	3 (2.0)		
Negative life events				
Yes	57 (38.0)	26 (17.3)	11.055	.001*^,^‡
No	61 (40.7)	6 (4.0)		

BMI = body mass index, EPQ-RSC = Eysenck Personality Questionnaire Revised Short Scale.

* Chi-square test.

† Mann–Whitney *U* test.

‡ *P* < .05.

**Table 4:** Univariate analysis of self-rating depression scale scores in patients with thyroid nodules.

Variables	SDS < 50 (No depression, n = 101, %)	SDS ≥ 50 (Depression, n = 49, %)	*χ*^2^/*Z*	*P*
Gender				
Male	13 (8.7)	6 (4.0)	0.012	.914*
Female	88 (58.6)	43 (28.7)		
Age (years)	45 ± 10.88	43.78 ± 10.361	-0.92	.357†
20–39	28 (18.7)	18 (12.0)	1.260	.262*
40–59	64 (42.7)	29 (19.3)	0.245	.621*
≥60	9 (6.0)	2 (1.3)	0.533	.465*
BMI (kg/m^2^)	22.85 ± 3.343	22.23 ± 4.073	-2.032	.042*^,^‡
Marriage				
Married	93 (62.0)	46 (30.7)	0.004	.950*
Others	8 (5.3)	3 (2.0)		
Level of education				
High school or below	21 (14.0)	31 (20.7)	2.185	.139*
Secondary and tertiary	33 (22.0)	9 (6.0)	20.882	.000*^,^‡
College degree or above	19 (12.6)	37 (24.7)	7.770	.005*^,^‡
EPQ-RSC				
Melancholic	22 (14.7)	13 (8.7)	0.416	.519*
Phlegmatic	23 (15.3)	3 (2.0)	6.383	.012*^,^‡
Choleric	32 (21.3)	20 (13.3)	1.215	.270*
Sanguine	24 (16.0)	13 (8.7)	0.136	.712*
Number of nodules				
Solitary nodule	28 (18.7)	17 (11.3)	0.764	.382†
Multiple nodules	73 (48.7)	32 (21.3)		
Nodular echogenicity				
Hypoechoic	60 (40.0)	29 (19.3)	0.001	.979†
Non-hypoechoic	41 (27.3)	20 (13.4)		
Components of nodules				
Mainly solid	54 (36.1)	23 (15.3)	0.563	.453†
Mainly cystic	47 (31.3)	26 (17.3)		
Boundary				
Clear boundary	81 (54.0)	40 (26.7)	0.644	.835†
Unclear boundary	20 (13.3)	9 (6.0)		
Aspect ratio				
<1	84 (56.0)	39 (26.0)	0.286	.593†
>1	17 (11.3)	10 (6.7)		
Sleeping disorders	6.74 ± 3.402	5.80 ± 3.062	-1.611	.107†
Yes	37 (24.7)	14 (9.3)	0.956	.328*
No	64 (42.7)	35 (23.3)		
Fatigue	6.74 ± 3.754	5.94 ± 3.843	-1.271	.204†
Yes	83 (55.3)	33 (22.0)	4.140	.042*^,^‡
No	18 (12.0)	16 (10.7)		
Negative life events				
Yes	47 (31.3)	36 (24.0)	9.685	.002*^,^‡
No	54 (36.0)	13 (8.7)		

BMI = body mass index, EPQ-RSC = Eysenck Personality Questionnaire Revised Short Scale.

* Chi-square test.

† Mann–Whitney *U* test.

‡ *P* < .05.

### 3.4. Analysis of risk factors affecting anxiety and depression in patients with TNs

**Figure 3.**
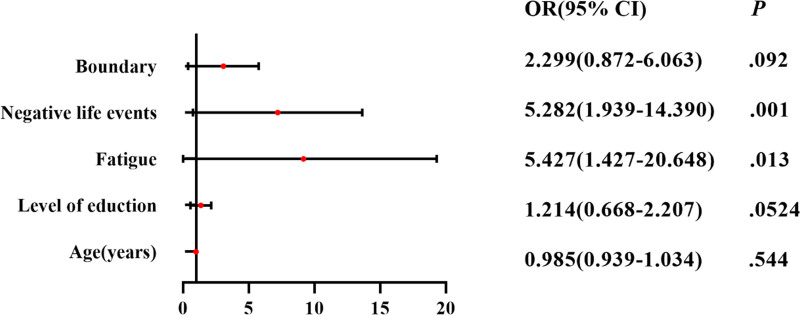
Binary logistic regression analysis of self-rating anxiety scale score in patients with thyroid nodules.

**Figure 4.**
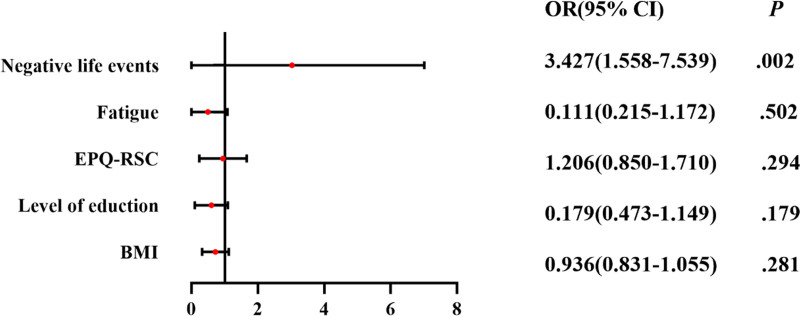
Binary logistic regression analysis of self-rating depression scale score in patients with thyroid nodules.

Binary logistic regression analysis of the significant factors in the univariate analysis revealed that fatigue status (OR = 5.427, 95% CI 1.427–20.648, *P* = .013) and negative life events (OR = 5.282, 95% CI 1.939–14.390, *P* = .001) were the independent influences on mood in patients with TNs. Negative life events (OR = 3.427, 95% CI 1.558–7.539, *P* = .002) were independent influences on depressed mood in TNs patients (Figs. 3 and 4). It is evident that negative life events are closely related to negative mood in TNs patients.

### 3.5. Percentage of negative life events in patients with TNs

**Figure 5.**
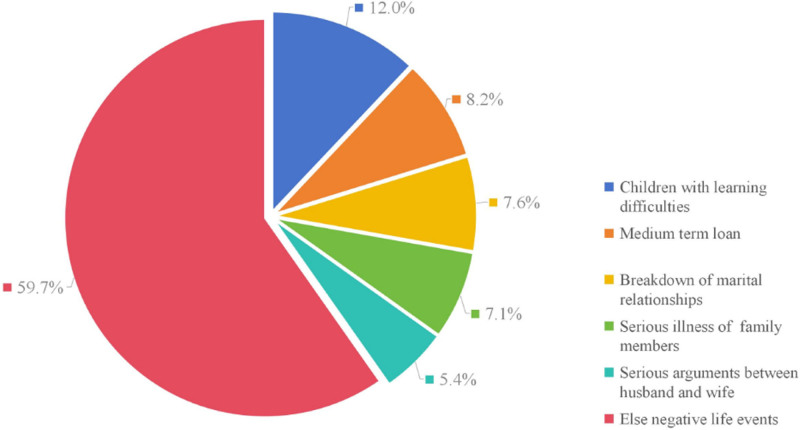
Percentage of the top 5 negative life events in patients with TNs.

The top 5 negative life events were children’s learning difficulties, medium-sized loans, breakdown of marital relationships, serious illness of family members, and serious arguments between husband and wife (see Table S1, Supplemental Digital Content, which illustrates the negative life events of patients with thyroid nodules, http://links.lww.com/MD/N968). The primary emphasis revolved around familial relationships, matrimony, and financial matters (Fig. 5).

## 4. Discussion

The occurrence of TNs is notably elevated throughout the Chinese population, with reported rates ranging from 10.12% to 46.56%.^[24]^ Furthermore, this condition exhibits distinct regional patterns.^[25]^ The objective of this study was to investigate the risk factors for negative emotions in patients with TNs. The findings indicated that patients with thyroid nodules have a higher risk of anxiety and depression in comparison to the control group. Moreover, an association was observed between thyroid nodules and the degree of anxiety and depression, which is consistent with previous findings.^[14]^ Patients with TNs experience a decline in their quality of life due to the compression of essential structures such as the trachea or esophagus, general discomfort in the neck region, and concerns related to appearance.^[26]^ Furthermore, it is worth noting that the condition of fatigue has an impact on individuals’ negative feelings.^[27]^ This observation holds true for the participants in the present study who were diagnosed with thyroid nodules. Individuals who possess a heightened level of literacy tend to exhibit greater knowledge and cognitive capacities, enabling them to effectively navigate obstacles and stressors, hence mitigating the occurrence of negative emotions.^[28]^ In addition, the results indicated that the clarity of patients’ nodal borders has an impact on their anxiety levels. Moreover, research conducted during the COVID-19 pandemic revealed a correlation between a patient’s nodal microcalcifications and their negative moods of anxiety and depression.^[29]^ Negative life events are a kind of psychological stressor that refers to various changes or difficulties individuals encounter in their everyday lives, such as exams, illnesses, or the loss of loved ones.^[30]^ In this study, these events are mainly related to family, marriage, and economic aspects and are closely related to the negative emotions of TNs patients.

TNs are affected by a variety of factors.^[1]^ Undoubtedly, in light of the progress and evolution of contemporary medical practices, healthcare professionals are progressively recognizing the influence of psychological conditions on the initiation, progression, and prognosis of thyroid diseases. Contemporary research indicates that adverse experiences play a prominent role in the development of psychopathology.^[31]^ The experience of prolonged negative life events can lead to the accumulation of chronic stress, which is a nonspecific systemic response that occurs when the body is exposed to various negative internal and external factors over an extended period of time.^[32,33]^ Chronic stress leads to the hyperactivation of the HPA axis,^[34]^ resulting in an elevated synthesis of corticotropin-releasing hormone in the hypothalamus. Corticotropin-releasing hormone serves as a signaling molecule that stimulates the pituitary gland to release adrenocorticotropic hormone,^[35]^ which is released into the peripheral circulation, subsequently reaching the adrenal cortex and eliciting the production of cortisol.^[36]^ It can lead to the manifestation of anxiety and depression.^[37]^ Furthermore, the extended hyperactivation of the HPA axis can result in the suppression of the HPT axis.^[38]^ High levels of cortisol inhibit the release of thyrotropin-releasing hormone, suppressing the secretion of thyroid-stimulating hormone by the pituitary gland,^[39]^ causing elevated susceptibility to anxiety and depression.^[15]^ Additionally, the HPA and HPT axes affect disorders of neurotransmitters such as 5-hydroxytryptamine and dopamine,^[40]^ which have been seen to be associated with the risk of developing psychiatric disorders such as anxiety and depression.^[41]^

However, the results of this study should be interpreted with caution, given several limitations. Firstly, it is important to acknowledge that the utilization of a small sample size, confined to 3 hospitals in the Shandong region, may introduce potential biases in the obtained results. Secondly, the present study posited a hypothesis suggesting that the occurrence of negative life events may be associated with anxiety and depression in patients with TNs, which lacks direct evidence. The subsequent research processes will apply laboratory indicators such as thyroid-stimulating hormone to continue the in-depth analysis. Lastly, the study’s findings regarding the causal relationship between thyroid nodules and anxiety and depression remain ambiguous. To further elucidate this relationship, longitudinal studies should be used to investigate whether individuals with anxiety or depression develop thyroid lesions over an extended period of time.

## 5. Conclusions

The results of the study offer a foundation for understanding the occurrence of negative emotions among Chinese patients with TNs. This study suggests that negative life events influence anxiety and depression in patients with TNs. Consequently, it is recommended that emotional screening and treatment be conducted for patients with TNs, and tailored psychological interventions be implemented based on the specific negative life events encountered. These measures aim to effectively address patients’ negative emotions and ultimately improve their prognosis.

## Acknowledgments

The authors would like to express their sincere gratitude to all the participants who took part in the survey, as well as to Xiyan Zhao from the Department of Foreign Languages, China University of Petroleum, for her help in writing the manuscript.

## Author contributions

**Conceptualization:** Mengxuan Wang.

**Data curation:** Guoshan Yuan, Lv-Ning Ren.

**Formal analysis:** Shuo Wang.

**Funding acquisition:** Mingzhou Gao, Dongmei Gao.

**Investigation:** Guoshan Yuan, Jieqiong Wang, Zhenhan Chu.

**Methodology:** Shuo Wang.

**Project administration:** Dongmei Gao.

**Resources:** Guoshan Yuan, Zhenhan Chu, Lv-Ning Ren.

**Software:** Shuo Wang.

**Validation:** Mingzhou Gao, Jieqiong Wang, Lv-Ning Ren, Dongmei Gao.

**Visualization:** Mengxuan Wang, Mingzhou Gao.

**Writing – original draft:** Mengxuan Wang.

**Writing – review & editing:** Mengxuan Wang, Dongmei Gao.

## Supplementary Material


